# Intra- and inter- observer reliability of anthropometric measurements and blood pressure in primary schoolchildren and adults: the Feel4Diabetes-study

**DOI:** 10.1186/s12902-020-0501-1

**Published:** 2020-03-12

**Authors:** Odysseas Androutsos, Costas Anastasiou, Christina-Paulina Lambrinou, Christina Mavrogianni, Greet Cardon, Vicky Van Stappen, Jemina Kivelä, Katja Wikström, Luis A. Moreno, Violeta Iotova, Kaloyan Tsochev, Nevena Chakarova, Tímea Ungvári, Zoltán Jancso, Konstantinos Makrilakis, Yannis Manios, on behalf of the Feel4diabetes-study group, Yannis Manios, Greet Cardon, Jaana Lindström, Peter Schwarz, Konstantinos Makrilakis, Lieven Annemans, Ignacio Garamendi, Odysseas Androutsos, Kalliopi Karatzi, Christina Mavrogianni, Konstantina Tsoutsoulopoulou, Christina Katsarou, Eva Karaglani, Irini Qira, Efstathios Skoufas, Konstantina Maragkopoulou, Antigone Tsiafitsa, Irini Sotiropoulou, Michalis Tsolakos, Effie Argyri, Mary Nikolaou, Eleni-Anna Vampouli, Christina Filippou, Gatsiou Katerina, Efstratios Dimitriadis, Tiina Laatikainen, Katja Wikström, Petteri Hovi, Jemina Kivelä, Päivi Valve, Esko Levälahti, Eeva Virtanen, Vicky Van Stappen, Nele Huys, Ruben Willems, Samyah Shadid, Ivonne Panchyrz, Maxi Holland, Patrick Timpel, Stavros Liatis, George Dafoulas, Christina-Paulina Lambrinou, Angeliki Giannopoulou, Lydia Tsirigoti, Evi Fappa, Costas Anastasiou, Konstantina Zachari, Lala Rabemananjara, Maria Stella de Sabata, Winne Ko, Luis Moreno, Fernando Civeira, Gloria Bueno, Pilar De Miguel-Etayo, Esther Mª. Gonzalez-Gil, Maria I. Mesana, Germán Vicente-Rodriguez, Gerardo Rodriguez, Lucia Baila-Rueda, Ana Cenarro, Estíbaliz Jarauta, Rocío Mateo-Gallego, Violeta Iotova, Tsvetalina Tankova, Natalia Usheva, Kaloyan Tsochev, Nevena Chakarova, Sonya Galcheva, Rumyana Dimova, Yana Bocheva, Zhaneta Radkova, Vanya Marinova, Yuliya Bazdarska, Tanya Stefanova, Imre Rurik, Timea Ungvari, Zoltán Jancsó, Anna Nánási, László Kolozsvári, Csilla Semánova, Remberto Martinez, Marcos Tong, Kaisla Joutsenniemi, Katrina Wendel-Mitoraj

**Affiliations:** 1 Department of Nutrition and Dietetics, School of Health Science and Education, Harokopio University, 70 El Venizelou Ave, 176 71 Kallithea, Athens, Greece; 2 Department of Nutrition and Dietetics, School of Physical Education, Sport Science and Dietetics, University of Thessaly, Trikala, Greece; 3 Department of Movement and Sports Sciences, Ghent University, Ghent, Belgium; 4 Department of Public Health Solutions, National Institute for Health and Welfare, Helsinki, Finland; 5 Universidad De Zaragoza, Zaragoza, Spain; 6 Department of Paediatrics, Medical University Varna, Varna, Bulgaria; 7 Department of Diabetology, Clinical Center of Endocrinology, Medical University Sofia, Sofia, Bulgaria; 8 Department of Family and Occupational Medicine, University of Debrecen, Debrecen, Hungary; 9 National and Kapodistrian University of Athens, Athens, Greece

**Keywords:** Obesity, Type 2 diabetes, Vulnerable, Children, Families

## Abstract

**Background:**

Feel4Diabetes was a large-scale, multicenter lifestyle intervention aiming to prevent type 2 diabetes among families from vulnerable population groups in six European countries (Belgium, Bulgaria, Finland, Greece, Hungary and Spain). The current study aimed to describe the process that was followed to harmonize and standardize the measurement of anthropometric (weight, height and waist circumference) and blood pressure (systolic and diastolic) indices, as well as to assess the intra- and inter- observer reliability of these measurements.

**Methods:**

A central training workshop was conducted prior to the baseline measurements of the Feel4Diabetes-intervention. One researcher from each intervention country, as well as 12 adults and 12 children (for the anthropometric measurements) and 21 adults (for the blood pressure measurements) participated in this workshop. Technical Error of Measurement (TEM) and reliability (%R) were calculated to assess the reliability of the indices which were assessed to evaluate the outcome of the Feel4Diabetes-intervention. The Feel4Diabetes-intervention is registered at https://clinicaltrials.gov/ (NCT02393872).

**Results:**

Intra-observer reliability was found to be higher than 99.5% for all anthropometric measurements in both children and adults. Inter-observer reliability was found to be higher than 98% regarding the anthropometric measurements, while for blood pressure measurements %R was 76.62 and 91.38% for systolic and diastolic blood pressure measurements, respectively.

**Conclusion:**

The central training of the Fee4Diabetes-intervention ensured that the data collected for the outcome evaluation of the Feel4Diabetes-intervention in the six European countries at three different time points (baseline, follow-up 1 and follow-up 2) were valid and comparable.

## Background

Type 2 diabetes is one of the major causes of morbidity and mortality [1]. Considering that a large segment of the population is undiagnosed, the actual prevalence of type 2 diabetes may be significantly higher than it is currently estimated [1]. Therefore, lifestyle interventions that can effectively tackle the risk factors for developing type 2 diabetes, such as obesity and obesity-related metabolic risk factors, are urgently needed.

The Feel4Diabetes-intervention was a school- and community- based intervention, aiming to promote healthy lifestyle and prevent type 2 diabetes among families from vulnerable population groups [2]. It was implemented in six European countries (Belgium, Bulgaria, Finland, Greece, Hungary and Spain), using standard procedures and protocols [2]. The effectiveness of the Feel4Diabetes-intervention will be evaluated regarding its impact, outcome, process and cost-effectiveness. Anthropometric (weight, height and waist circumference) and blood pressure data, as well as blood samples were collected by the local research staff in the six countries participating in the Feel4Diabetes-intervention to evaluate its outcome.

It is of great importance that multicenter studies, such as Feel4Diabetes, use harmonized and standardized measurement procedures, as well as reliable and valid tools to assess their effectiveness, in order to reduce the risk of systematic bias due to deviations of the research staff from the study protocol [3]. Previous similar, large-scale studies, such as the ToyBox-study, the IDEFICS-study and the WHO Multicentre Growth Reference Study, have assessed the intra- and inter- observed reliability of the measurements taken [4–6]. Reliability reflects the degree to which the variability of measurements is attributed to parameters other than the measurement error, with intra-observer reliability referring to repeated measurements taken on the same subject by the same examiner and inter-observer reliability referring to repeated measurements taken on same subjects by different examiners [4]. Higher values of intra- and inter- observed reliability indicate higher precision of measurements taken by each examiner or the research staff members, accordingly [4].

The aim of the present study was to describe the harmonization and standardization process and assess the intra- and inter- observer reliability for the anthropometric and blood pressure measurements conducted in the Feel4Diabetes-intervention and used to evaluate its effectiveness.

## Methods

The protocol of the anthropometric and blood pressure measurements, which was developed to standardize and harmonize the procedures followed in the baseline, follow-up 1 and follow-up 2 measurements of the Feel4Diabetes-intervention, is described elsewhere (Androutsos et al., under review).

A central training workshop was held in Ghent (Belgium) prior to the baseline measurements (i.e. September 2015), in order to train representative researchers from the six countries participating in the Feel4Diabetes-intervention on all assessment tools and methods. The representative researchers were the same measuring on the field or the ones who were responsible for training the research staff in their country. The measurement results from the workshop were used to assess researchers’ intra- and inter- observer reliability regarding the anthropometric (weight and height for both adults and children, and waist circumference for adults) and blood pressure (systolic and diastolic, for adults) measurements. Following a theoretical introduction, a practical training was implemented, and thereafter, intra- and inter-observer reliability was assessed. Data were recorded and reliability (%R) was calculated, as described below.

Six researchers (one from each intervention country), as well as 12 adults and 12 children (for the anthropometric measurements) and 21 adults (for the blood pressure measurement) participated in the present study. All subjects (adults and parents of children) signed an informed consent form before enrolling in the study, while children also assented orally before being measured. Children and adults from all weight categories (i.e. normal weight, overweight or obese) were included. The type of equipment used in this study was the same as the type that was used in the baseline, follow-up 1 and follow-up 2 measurements of the Feel4Diabetes-intervention. More specifically, weight was measured with electronic scales (SECA 813), height with a portable stadiometer (SECA 217), waist circumference (WC) with a measuring tape (SECA 201) and blood pressure with an electronic monitor (OMRON M6 AC). All equipment was calibrated before the conduct of this study.

All anthropometric measurements were taken twice by dyads of researchers. A third measurement was also taken, in the case that the previous two measurements differed >100 g for weight, > 1 cm for height or > 1 cm for waist circumference. Subjects were asked to remove their shoes, heavy outer garments, hair ornaments, jewellery, head dress from the top of the head and heavy clothing (e.g. jackets), as well as to empty their bladders before the measurements. Moreover, they were asked to stand still, at an erect position during the measurements. Prior to the measurement of height, subjects’ head was placed in the Frankfort plane. For the measurement of waist circumference, the measuring tape was placed horizontally, midway between the lowest rib margin and the iliac crest. Weight was recorded to the nearest 0,1 kg and height and waist circumference to the nearest 0,1 cm, at the end of a gentle exhalation.

For the measurement of blood pressure (systolic and diastolic) subjects were asked to abstain from eating, drinking, smoking and heavy exercise for at least 1 h before the measurement, as well as to empty their urinary bladder, remove any clothes or other material from their arms and sit relaxed for 5 min on a chair. During the measurements they were asked to sit still and relaxed, as well as to keep their arm at the level of their heart. Measurements were taken twice on the right arm. Appropriate cuff was selected, according to subjects’ arm size. Between the two measurements a period of 2–3 min was allowed.

To assess the intra- and inter- observer reliability for the anthropometric and blood pressure measurements ‘Technical Error of Measurement’ (TEM) was calculated based on the following formula:

$$ \mathrm{TEM}=\sqrt{\left(\right[\sum \mathrm{n}\ \left\{\left(\sum \mathrm{K}\ \mathrm{M}2\right)-\left(\left[\sum \mathrm{K}\ \mathrm{M}\right]2/\mathrm{K}\left\}\right]\right)/\mathrm{n}\left(\mathrm{K}-1\right)\right)} $$

For the intra-observer reliability of each method (i.e. weight, height, waist circumference), TEM was calculated using data obtained from three consecutive measurements taken on each subject (child or adult) by each researcher separately. For the inter-observer reliability TEM was calculated using data obtained from measurements taken on each subject by each researcher. Moreover, R as a percentage (%R) was calculated based on the formula R% = 1− (total TEM^2^/SD^2^). All statistical analyses were performed using SPSS version 20.0 (IBM Corp, Armonk, NY, USA).

## Results

Table 1 shows the results of the intra-observer reliability of the anthropometric measurements in both children and adults. Regarding adults’ intra-observer reliability, TEMs ranged between 0.08–0.27 cm for height, 0.04–0.12 kg for weight and 0.20–0.47 cm for WC, while in children TEMs ranged between 0.06–0.14 cm for height and 0.04–0.07 kg for weight. Considering the data from all six researchers together, intra-observer reliability (%R) was above 99.5% for all anthropometric measurements in both children and adults.

**Table 1:** Intra-observer reliability of anthropometric measurements in children and adults. Feel4Diabetes-study

	Adults				Children			
	n	SD	TEM	%R	n	SD	TEM	%R
Belgium								
Height (cm)	12	6.72	0.10	99.98	12	5.78	0.14	99.94
Weight (kg)	12	11.90	0.12	99.99	12	2.81	0.07	99.94
Waist circumference (cm)	12	11.89	0.46	99.85	–	–	–	–
Bulgaria								
Height (cm)	12	6.87	0.11	99.87	12	5.70	0.08	99.98
Weight (kg)	12	11.93	0.06	100.00	12	2.83	0.05	99.97
Waist circumference (cm)	12	12.75	0.47	99.97	–	–	–	–
Finland								
Height (cm)	12	6.75	0.10	99.98	12	5.70	0.10	99.97
Weight (kg)	12	11.89	0.05	100.00	12	2.80	0.04	99.98
Waist circumference (cm)	12	11.69	0.20	99.97	–	–	–	–
Greece								
Height (cm)	12	6.75	0.17	99.94	12	5.79	0.13	99.95
Weight (kg)	12	11.95	0.04	100.00	12	2.82	0.07	99.94
Waist circumference (cm)	12	12.43	0.31	99.94	–	–	–	–
Hungary								
Height (cm)	12	6.70	0.08	99.98	12	5.70	0.13	99.95
Weight (kg)	12	11.96	0.07	100.00	12	2.84	0.06	99.96
Waist circumference (cm)	12	11.21	0.12	99.99	–	–	–	–
Spain								
Height (cm)	12	6.76	0.08	99.98	12	5.73	0.06	99.99
Weight (kg)	12	11.95	0.06	100.00	12	2.80	0.04	99.98
Waist circumference (cm)	12	11.65	0.27	99.95	–	–	–	–
All countries								
Height (cm)	72	6.68	0.27	99.83	72	5.67	0.27	99.77
Weight (kg)	72	11.79	0.18	99.98	72	2.78	0.14	99.76
Waist circumference (cm)	72	11.85	0.81	99.53	–	–	–	–

%*R* Relative coefficient of reliability, *TEM* Technical error of measurement

Table 2 shows the inter-observer reliability results for the anthropometric measurements in both children and adults and the blood pressure measurements in adults. Regarding anthropometric measurements, in adults TEMs were 0.29 cm for height, 1.49 kg for weight and 2.5 cm for WC, while in children TEMs were 0.27 cm for height and 0.06 kg for weight. Inter-observer %*R* was above 98% for all anthropometric measurements. Concerning blood pressure measurements, inter- observer %*R* was 76.6 and 91.4% for systolic and diastolic blood pressure measurements, respectively.

**Table 2:** Inter-observer reliability of anthropometric measurements and blood pressure measurements in children and adults. Feel4Diabetes-study

	Adults				Children			
	n	SD	TEM	%R	N	SD	TEM	%R
Height (cm)	12	6.68	0.29	99.82	12	5.67	0.27	99.78
Weight (kg)	12	11.79	1.49	98.41	12	2.78	0.06	99.96
Waist circumference (cm)	12	11.85	2.50	95.56	–	–	–	–
Blood pressure								
Systolic (mm Hg)	21	11.76	5.69	76.62	–	–	–	–
Diastolic (mm Hg)	21	7.45	2.18	91.38	–	–	–	–

%*R* Relative coefficient of reliability, *TEM* Technical error of measurement

## Discussion

A series of harmonization and standardization procedures were conducted, aiming to increase the quality and comparability of data to be collected across the six countries participating in the Feel4Diabetes-intervention. To assess the intra- and inter- observer reliability for the anthropometric (weight, height, waist circumference) and blood pressure (systolic and diastolic) indices which were selected to evaluate the effectiveness (outcome evaluation) of the Feel4Diabetes-intervention, TEM and R (%R) were calculated, using data obtained from the current, preparatory study. These indices (TEM and %R) are widely used in the literature to assess the intra- and inter- observer reliability [7, 8].

According to the findings of the present study, the researchers from the six intervention countries achieved a very good intra- and inter-observer agreement before performing the fieldwork (baseline, follow-up 1 and follow-up 2 measurements) in the main study. More specifically, intra-observer reliability was found to be ‘excellent’ for all anthropometric measurements, in both children and adults, as it was above 99.5%. Inter-observer reliability was also found to be ‘excellent’ regarding the anthropometric measurements (%*R >* 98%), while in blood pressure measurements %*R* was 76.6 and 91.4% for systolic and diastolic blood pressure measurements, respectively, which are considered ‘very good’.

In large-scale, multicenter studies such as the Feel4Diabetes-study it is of outmost importance to standardize the measurement procedures across all participating countries and centers and collect valid and comparable data, which will guide future research and public health priorities. Although not all previous similar studies have reported their standardization procedures and studies, some recent studies reported the intra- and inter- observed reliability of the measurements conducted [3–7, 9]. More specifically, regarding children’s anthropometric indices, the ToyBox-study reported that intra- and inter- observer reliability for preschool children’s weight and height was “excellent” (%R ≥ 98%), while for their waist circumference it was %R ≥ 92% [4]. Similarly, the IDEFICS-study showed that intra- and inter- observer reliability for weight, height and waist circumference in children aged 2–9 was “excellent” (%R ≥ 99%) [5]. Moreover, in the WHO Multicentre Growth Reference Study %R was found to be higher than 95% for height [6]. In the HELENA-study, which focused on adolescence, the intra- and inter- observer reliability for waist circumference was found to be %R ≥ 90% [10]. Furthermore, the AVENA-study, which also focused on adolescent populations, reported that TEM for waist circumference was less than 1 mm and %R was >95%, while another review study reported that inter-observer reliability (%R) for waist circumference varies between 86 and 99% [7, 11]. The values reported by these multicenter cohorts were similar to those observed in the Feel4Diabetes-study.

The findings of the present study should be interpreted under the light of its strengths and limitations. The intra- and inter- observer reliability assessments conducted in this study were based on data obtained from population groups that were similar to those of the Feel4Diabetes-intervention in means of age groups (i.e. children attending first grades of primary school and adults), demographic characteristics (i.e. families from low-socioeconomic areas) and weight categories (i.e. normal weight, overweight or obese). Moreover, the same protocols, procedures and types of equipment for the measurement of the anthropometric indices and blood pressure were used in this study and in the Feel4Diabetes-intervention. On the other hand, it was not feasible to repeat the intra- and inter- observer reliability assessments during the implementation of the Feel4Diabetes-intervention, due to time and budget restrictions. Standardization of techniques prior to the trial may not guarantee that the same precision will be in place throughout, however this procedure is commonly followed in large-scale studies such as the HELENA-study and the ToyBox-study [4, 10]. Moreover, the research staff that was trained in the central training was part of the staff that actually performed the baseline and follow-up measurements. It was also aimed that the research staff in each intervention country remained the same in all time points that data were collected. These conditions may have reduced possibilities of error.

## Conclusions

The central training of representative researchers based on standard protocols and procedures, which was conducted prior to the main study, ensured that the data collected at baseline, follow-up 1 and follow-up 2 in the Feel4Diabetes-intervention were valid and comparable. Intra- and inter- observer reliability of all anthropometric measurements was found to be “excellent” (%R ≥ 95%), while inter- observer for blood pressure measurement was “very good” (%R ≥ 75%).

## Data Availability

The datasets generated and/or analyzed during the current study are not publicly available, since the data used is confidential based on Feel4Diabetes publications rules, but are available from the corresponding author on reasonable request.

## Abbreviations

R: Reliability
TEM: Technical Error of Measurement
